# Factors that determine the connectedness with nature in rural and urban contexts

**DOI:** 10.1371/journal.pone.0309812

**Published:** 2024-08-30

**Authors:** Luis Macias-Zambrano, Esther Cuadrado, Antonio J. Carpio

**Affiliations:** 1 Department of Zoology, University of Córdoba, Cordoba, Spain; 2 Life Sciences and Technologies Faculty, Lay University Eloy Alfaro de Manabí, Manta, Ecuador; 3 Department of Psychology, University of Cordoba, Cordoba, Spain; 4 Maimonides Biomedical Research Institute of Cordoba (IMIBIC), Cordoba, Spain; 5 Department of Botany, Ecology and Plant Physiology, Research Group on Education and Biodiversity Management (GESBIO), University of Cordoba, Cordoba, Spain; Tsinghua University, CHINA

## Abstract

Connectedness with nature is considered a key element for the future of conservation. There are both internal and external factors that determine the levels of connectedness with nature. Among these factors are gender, age, knowledge about the environment and place of residence. In the latter case, there may be differences in how urban and rural dwellers perceive nature, based on their experiences and contact with it. The main objective of this research is to evaluate and establish the factors that influence and determine the levels of connection with nature, examining how these factors relate and interact with each other, taking the urban and rural context as starting point. The ABC-CNS scale, which addresses the affective, behavioural and cognitive aspects of the connection with nature, was applied via online questionnaire to a sample of university students from two countries, Spain (496 students) and Ecuador (872 students), who were also clustered according to career, age, gender, and place of residence. The results obtained through four General Linear Mixed Models (ABC-CNS and its dimensions as response variables) and LSD test, demonstrated that the ABC-CNS scale presented significant differences for all the variables analyzed (place of residence, gender, age, and career), also demonstrating which levels influence and interact in higher ABC-CNS values. Finally, the study concludes that the analyzed factors contribute to the development of the connection with nature. In the case of place of residence, attention should be given to the specific settings of the environments under study.

## Introduction

The increase in the urban population, along with the increased use of new entertainment technologies, has caused people to spend less time outdoors in nature [1]. This reduction of contact with nature is considered one of the reasons why society is increasingly disconnected from today’s conservation problems. Various studies have shown that the individual’s attachment to nature, known as connection to nature, positively influences the development of environmental behaviours, attitudes, and concerns [2–4].

The nature connectedness is considered a critical factor for the future of conservation [1] In fact, this connection may be more important for encouraging pro-environmental behaviour than merely accepting an ecological paradigm based on beliefs [5]. Connection with nature is greater in people who have had previous experiences in natural environments [6]. Considering nature connectedness as a potential precursor to perceiving positive experiences in natural settings [7], individuals positively linked to a certain location are more likely to exhibit positive behaviours [8], and care more about conservation [1]. Nature connectedness describes a deep appreciation and affiliation toward natural environments. Therefore, individuals with high levels of nature connectedness are assumed to perceive natural settings as more attractive and fascinating [7], showing a positive appreciation towards for the natural environment over the built environment [6].

The gap between pro-environmental attitudes and behaviour is influenced by the knowledge and experience gained from the level of personal exposure to natural environments, as well as by the general perception of the culture of natural resources in the community [9]. Internal and external demographic factors, including sociocultural, economic and institutional factors are considered influential in environmental behaviour [10].

Gender and age are internal demographic factors that influence nature connectedness [2]. Gender plays a critical role in understanding how people incorporate nature into their cognitive representation of themselves [11]. Previous studies have been able to demonstrate significant differences between genders, revealing a deeper connection with nature in the female gender [2, 11], but leaving room for the analysis about the gender influence in the affective, cognitive and behavioural dimensions of connectedness with nature. In the case of age, it has been shown that levels of nature connectedness can increase or decrease with age, depending on the context [12]. The literature reviewed has shown that although age influences the connectedness with nature, revealing the importance of fostering connectedness with nature in children [13], not much effort has been put into analyzing this connection in young adults.

According to Ernst & Theimer [13], knowledge is key in the connection with nature. Education through contact with nature, using experiences and outdoor practices, affects the development of environmental concerns and preference towards natural environments. Increases in levels of nature connectedness have been achieved through education, even in higher education [14]. Although education is important in the development of the connection with nature, as happens with age, many of the studies focus on the early ages of the individual [14]. In addition, most studies focus on specific environmental education programs, leaving aside the knowledge acquired through the curriculum in university education.

Place of residence is another important factor in explaining nature connectedness and pro-environmental behaviour [15]. People who feel attached to natural environments develop a sense of identity with that environment [16]. Also, affection and knowledge about a place increase the likelihood of protective behaviours toward that place and can generate a sense of commitment and responsibility towards the places which the individual feels more linked [8]. Contact with nature can result in high levels of nature connectedness. This can occur when an individual interacts with natural components or is surrounded by a natural environment. These interactions are diverse, from outdoor sports, such as cycling or walking, to working in an office overlooking the forest [17].

Studies developed in urban and rural environments have shown differences in how individuals perceive the natural environment; these perceptions may also be shaped by the cultural context in which they develop [18]. Several studies report that exposure to nature is associated with its proximity and the availability of green spaces in the living environment, especially in highly urbanized areas [6]. Contact with natural environments contributes to the development of experiences that promote well-being and health. Conversely, inequality in access to healthy natural environments between individuals of different ethnicities and socioeconomic backgrounds leads to disparities in development [18]. As a result, the way rural dwellers experience the environment differs from their counterparts in urban areas [19]. In these contexts, the level of urbanization and environmental degradation to which individuals are exposed can also play an important role [20]. Scientific literature states that rural and urban populations differ in their environmental and ecological perceptions [21]. Since most of the previous literature coincides with the malleability of the elements and factors that can converge in urban and rural settings, resulting in different and particular context shaping the connectedness with nature, this left an open door for research to support with findings centered in local environments and realities.

The rapid pace of urbanization has significantly altered the relationship between individuals and nature, impacting well-being and societal harmony. Understanding connectedness with nature is crucial due to its profound implications for quality of life. Identifying the factors shaping nature connectedness provide critical insights for policymakers, urban planners, and health professionals. For example, Restall et al. [22] highlight the importance of the nature connectedness in the management of protected areas, especially when involving communities in their governance. It has been reported that higher levels of nature connectedness correlate with a greater desire to get involved in the management of these areas.

The main objective of this research is to assess and establish some factors that influence and explain levels of nature connectedness and its dimensions, examining how these factors relate to and interact with each other, with a focus on urban and rural contexts. In addition, the following hypothesis is raised: the individuals place of residence influences the levels of nature connectedness. This study is novel in its comparative analysis of university students’ connectedness with nature across different cultural contexts, specifically Spain and Ecuador. Additionally, this research employs the ABC-CNS scale for a comprehensive understanding of how affective, behaviour, and cognitive dimensions are influenced by internal and external factors. By integrating these dimensions, this study offers new insights into the role of culture in shaping environmental attitudes and behaviours among university students.

## Material and methods

### Participants

The present research was carried out with a sample composed of university students from two countries: Spain (496 students) and Ecuador (872 students), resulting in a total of 1,368 individuals (932 women and 436 men) with a mean age of 21.09 years (standard deviation of 3.78), ranging from 18 to 49 years old. This sample was drawn from classes where the researchers were teaching at the time of this research. The sample was selected taking into consideration the careers that offer and/or represent different levels of knowledge and contact with nature. Also, the sample comprises university students living in rural or urban zones near the cities where the universities’ campuses are located. The sample is not representative of the broader student population, but representative of the careers included in the research. Students were clustered according to the careers in which they were enrolled and by place of residence.

### Procedure

This study, which was previously approved by the Cordoba Research Ethics Committee, through code 4429, was correlational and transversal. University professors disseminated the link to the online questionnaire on the websites of the courses they were teaching and invited their students to participate in a study about perception about the natural environment. For informed consent, they were advised that participation was voluntary and anonymous, and that they could withdraw whenever they wanted. Before responding, the participants gave their consent for the respective data collection by checking their approval on an item in the questionnaire that states “I give my consent to participate in the study” (Doy mi consentimiento para participar en el estudio). The data analyzed only correspond to students that checked their consent in the mentioned item, also, no minors were enrolled in the study. The data gathering took place between February 3 and March 27, 2020.

### Measures

A questionnaire was designed to collect basic information such as age and gender, as well as variables as country, university, career, place of residence. The ABC Connectedness with Nature scale (ABC-CNS) was used for the nature connectedness variable [23]. This scale is focused on the analysis of the nature connectedness as an attitude, based in a tridimensional model that aboard the affective, behavioural, and cognitive aspects of the construct. Each dimension is composed of five items valued on a five-point Likert scale. The reliability of this scale was *α* = 0.949. The ABC-CNS scale was previously validated by the authors of the original scale through an Exploratory Factor Analysis using Promax rotation and Confirmatory Factor Analysis with MPLUS [23]. Previous analyses were carried out using SPSS statistics software.

Cronbach’s alpha was carried out using SPSS statistics software to know determine the reliability of the ABC-CNS scale and its dimensions in the research sample. The result for the ABC-CNS scale was .949 (Spain .919, Ecuador .961); for the dimensions were: Cognitive .941 (Spain .890, Ecuador .962), Affective .938 (Spain .886, Ecuador .960) and Behaviour .872 (Spain .748, Ecuador .922). As we can see there was optimum values for the scale, as well present in both countries. Also, for the dimensions all except Behaviour are over .900, and by country Ecuador was over .900 in all dimensions.

### Data analysis

In order to determine the underlying intrinsic and extrinsic factors driving the nature connectedness, four General Linear Mixed Models (GLMMs) were performed using the variable ABC global (model 1), affective (model 2), behavioural (model 3), and cognitive dimensions (model 4) as response variables. These variables were calculated as the average of the items that comprise each dimension, and for ABC global, the average of each item. A protocol for data exploration was applied and the assumptions were checked on the residuals of the model [24]. In all models, a normal distribution and the identity link function were used. In these models, gender (2 levels), age (three levels), career (three levels), place of residence (three levels), and the interaction between gender and place of residence were included as fixed effect. The university nested within the country was included as a random factor. Fisher’s least significant difference test (LSD test) for comparisons of the estimated means within a mixed analysis was developed to check the differences among the levels of categorical variables. Statistical analyses were performed by employing InfoStats software.

## Results

### Descriptive analysis

The mean score for the ABC-CNS scale was 4.08 (± SD = 0.76) on a five-points scale. The means for the dimensions were: Cognitive 4.00 (± SD = 0.95), Affective 4.22 (± SD = 0.87), Behaviour 4.02 (± SD = 0.77). For the scale and its dimensions, means values in Ecuador were above 4.00, in contrast, in Spain values are below 4.00, except for Affective dimension. In Spain, mean scores above 4.00 were observed in variables such countryside, age range 26–50, and in environment and biology careers. In Ecuador, all variables displayed mean values above 4.00, with higher scores on women, age range 26–50 and environment and biology careers. The detailed sample is shown in Table 1. Details about descriptive analysis by each variable and country are in S1 Table. Also, descriptive analysis for each item on the scale is on S2 Table.

**Table 1:** Place of residence, gender, age, university, and career of the participants.

		Spain	Ecuador	Total
Residence	City	319	530	849
	Town	163	175	338
	Countryside	14	167	181
Gender	Men	112	324	436
	Women	384	548	932
Age range	18–20	277	487	764
	21–25	186	315	501
	26–50	33	70	103
University	ESPAM	0	167	167
	ULEAM	0	286	286
	UTM	0	334	334
	UNESUM	0	85	85
	USAL	25	0	25
	UCO	471	0	471
Career	Engineering, industry and administration	0	300	300
	Environment and biology	103	278	381
	Psychology, education and social sciences	393	294	687

### Factors that determine the connection with nature

Regarding the variables that affect the nature connectedness, Model I, the ABC-CNS scale, presented significant differences for all the variables analyzed (Table 2). Model II (Cognitive) presented significant differences in all the variables except career. Model III (Affective dimension) did not present significant differences for age range and place of residence. Finally Model IV (Behavioural dimension) only presented significant differences for age range and career (S3 Table).

**Table 2:** General Linear Mixed Model (GLMM) with the variable ABC global (model 1), as response variable.

		Marginal			
		F-value	p-value		Value ± S.E.
**MODEL I**	Intercept	1161.49	<0.0001	Intercept	3.93 ± 0.14
**ABCCNS**	Gender	11.40	0.0008	Women	0.11 ± 0.05
	Age range	6.77	0.0012	Age range 21–25 Age range 26–50	0.01 ± 0.05 0.28 ± 0.08
	Career	4.05	0.0176	Environment and biology Psychology. education and social sciences	0.12 ± 0.07 -0.04 ± 0.07
	Place of residence	3.11	0.0447	Countryside Town	0.11 ± 0.11 -0.24 ± 0.09
	Gender:Place of residence	4.00	0.0186	Women:Countryside Women:Town	-0.08 ± 0.13 0.28 ± 0.11

Reference levels

Gender: Men

Age range: 18–20

Career: Engineering. industry, and administration

Place of residence: City

The post-hoc Fisher test (ABC-CNS in Table 3, rest of the factors in S4 Table) shows the following findings. The variable gender presented significative differences between the two levels in all models except of Model IV, with higher values for women. Regarding age range, in all models, the range 26–50 years old stands as an independent category from the other two ranges, with this category also presenting higher values. The variable career presented the levels “environment and biology” and “psychology, education and social sciences” as separated categories, with the first one presenting higher value than the rest, although the level “engineering, industry, and administration” pertains to both previous categories, these findings apply to all models.

**Table 3:** LSD Fisher test (ABC-CNS). Mean values with a common letter are not significantly different (p > 0,05).

		ABC-CNS			
		Mean	S.E.		
Gender	Women	4.22	0.08	A	
	Men	4.04	0.08		B
Age range	26–50	4.32	0.10	A	
	21–25	4.04	0.08		B
	18–20	4.03	0.07		B
Career	Environment and biology	4.22	0.08	A	
	Engineering, industry and ..	4.12	0.09	A	B
	Psychology, education and ..	4.05	0.08		B
Residence	Countryside	4.22	0.09	A	
	City	4.14	0.07	A	
	Town	4.03	0.08		B
Gender:Residence	Women:Countryside	4.24	0.10	A	
	Women:Town	4.23	0.08	A	
	Men:Countryside	4.20	0.12	A	
	Women:City	4.20	0.08	A	
	Men:City	4.08	0.08	A	
	Men:Town	3.84	0.11		B

In the case of the variable place of residence, the difference among levels varies between the models. For the Model I, countryside and town stands in their own categories, while city fits in both previous categories. For Model II and III, both countryside and city made one category, while town made a category by itself. Countryside category values stay higher than the other categories. Finally, for the combination of the variables place of residence and gender, like the previous variable, place of residence, differences among levels appeared between the models. For Model 1, from the six levels, only men:town appeared in its own category, the five levels left join together in one category, this effect is present also in Model II.

In Model III, the distinctions become more complex. The levels women:town, men:city, and men:town each stand in their own categories. However, women:countryside, women:city, and men:countryside share categories with both women:town and men:city. This results in men:town being the only level that does not show similarities with other levels. Additionally, the combination of women:countryside had the highest value across all models except Model IV. In Model III, it tied with women:town. In Model IV, the category of men:countryside presented the highest values.

## Discussion

Regarding our main objective, it was verified that factors such as gender, age, career (knowledge), and place of residence explain the levels of connectedness with nature. The findings indicate that the construct of connectedness with nature is indeed influenced by an amalgamation of both external and internal factors that shape it. Although many of these factors are inherent and cannot be modified, we can learn from their characteristics to develop more effective ways to enhance our connection with nature and improve our environmental behaviour.

Prior studies have explored the complex interplay between individuals and their environments. Mayer and Frantz [25] emphasize the positive impact of nature exposure on the developing of environmental attitudes, addressing the potential influence of limited natural spaces in urban areas on hindering connectedness. Also, Kellert and Wilson [26] found that direct interaction with nature, such as outdoor activities, fosters a deeper connection, particularly in rural settings. Kaplan [27] points out the mental restorative benefits of natural environments compared to the potential stressors in built settings. Our study aligns with the previous context identifying challenges in both, rural and urban environments, where built structures and other factors may difficult connectedness with nature, indicating a potential urban-rural disparity in the factors influencing nature connectedness. Regarding this potential disparity, Nisbet et al. [28] noted obstacles to nature connection due to the absence of natural spaces in environments.

Our research findings show correlations between age and connectedness to nature, that extents to the cognitive and behaviour dimensions. Many researchers support the relationship between age and connectedness to nature [2, 29, 30]. Although most of the studies focused on this relationship in children [2, 29], those results support the fact that age is an important predictor of connectedness to nature and thus may inluence environmental behaviour, more over in a context of contact with nature [31]. Results in our research shows that 26–50 age range had a strong difference compared to the other groups. Findings in research from Mustapa et al. [2], Liefländer et al. [31], van Heezik et al. [32] and Savolainen [33] show strong values of connectedness with nature in youngers groups, that seems to support the idea that connectedness with nature correlates negatively with age, especially in childhood. It is noteworthy that our context consists of adult students aged 18 and older, and it is not a comparison between older and younger groups. Based on the previous the age variable leads to other factors linked to the growth process, for example adolescence and learning. Liefländer et al. [31] mentions that during the transition towards adolescence there is a reduction in the level of connection with nature. This can explain the loss of connectedness with nature in adults. In our research, learning and knowledge may explain the differences between groups, given the context in which the study was conducted. In our study the knowledge and learning factors are attached to the career variable, that will be addressed later this section. Also, Passmore et al. [34] explains that findings like this are consistent with the “teenage dip”, so levels of nature connectedness do not reach preteen levels again until close to age 30 years. Kural et al. [12] found a positive correlation between age and connection with nature in a group of teachers that were in the age range of 31–35, as the age of teachers increases, their connectedness to nature scores increases, this may suggest that their relationships with nature develop based on experience. Also, Teixeira et al. [35] found in their research that increasing age contributed to a higher connectedness to nature, adding the higher connectedness to nature in older individuals is linked to a higher number of visits to natural spaces and to greater opportunities for engaging with them.

As previously mentioned, our study considered the variable career into the analysis. It was found that career has influence on connectedness with nature, also in its dimensions affective and behavioural. In the present research the career variable groups together a set of careers in common, based on the area of knowledge, with environment and biology group resulting in higher levels of connectedness with nature than the rest of the groups. We can assume, since our sample is composed of university students, that knowledge about environment is related, in greater or less extent, with the career they were enrolled. People knowledge is associated with the level of education [36]. For Winfried et al. [37] knowledge about environment can be achieve though education, adding that this knowledge is an important part of biological studies, together with practical learning with activities in nature. Richardson et al. [38] reports that people in environmental careers spend more time in nature, that could result in higher interest about nature and levels of connectedness. Knowledge about environment is positively correlated with connectedness with nature [37, 39, 40]. In this line, Bruni et al. [41] mentions the lack of knowledge about the local environment could be a factor to disconnection from nature. Prévot et al. [36] adds that knowledge correlates with environmental attitudes and behaviour, but although is necessary, is not sufficient, since it can increase connectedness to nature in short term. In this sense, Cuadrado et al. [42] found in an experimental study that broader disciplinary concentration related to environmental problems leads University students to behave in more pro-environmental and less selfish ways. For Otto & Pensini [43] acquiring knowledge of the functioning of the natural environment may, for example, confront individuals with the interconnectedness of all life, impacting their connectedness to nature.

Our findings also show significative differences between sex in connectedness with nature, and the dimensions cognitive and affective, with higher values in women than men. Pérez-Ramírez et al. [11] found gender is one of the determinant factors in the connectedness with nature. In the same way for Winfried et al. [37] gender is a factor that has a special influence on several environmental variables, as a strong predictor for environmental behaviour and attitude. High values of connectedness with nature in women are often reported in various contexts [11, 44]. Musitu-Ferrer et al. [45] mentions that, in relation to gender, much research has concluded that women show greater connectedness and empathy with the natural environment, commitment to environmental protection and more pro-environmental behaviours than men. Also, this can be explained through gender-role socialization. Socialization theory posits that individuals are shaped by gender expectations within the context of cultural norms, these differences in socialization between the genders could then be reflected in their environmentalism [46]. For Musitu-Ferrer et al. [45] the gender-socialization results in female’s heightened capacity for empathy what ends in greater commitment and empathy toward the natural environment. Musitu-Ferrer et al. [45] adds that women’s socialization is more oriented towards aid and cooperation and more pro-social and empathic, in turn implying greater empathic concern for other animals and nature in general. However, some studies report contradictory findings showings no differences between genders [29, 47, 48]. In our findings we report a difference in the interaction between gender and place of residence, resulting in the combination of men-town as a stand-alone group (with the lowest value), while the rest combinations grouped all together in one. In part, this result supports the previous reports, there are no differences between genders, and those differences found are more based in the context of the research. Teixeira et al. [49] addresses the differences between genders about proenvironmental behaviour, entails those differences to values influenced by social roles, these values can develop into positive environmental attitudes. Continuing, the author mentions these differences can be found depending on the dimensions analyzed. In the other hand, Teixeira et al. [35] mentions this lack of differences between genders could be more contextual, like availability of green space in the living area, and equality in the opportunity to connect with those natural spaces.

Finally, the place of residence variable reports influence for connectedness to nature, also, in the cognitive dimension. The findings show higher values for countryside in connectedness to nature and for all dimension for Spain, and for behaviour dimension in Ecuador, while city shows higher values in connectedness to nature, for affective and cognitive dimensions for Ecuador. Various authors have reported the influence that the place of residence variable has on the connection with nature [50]. As Boiral et al. [51] mentions, the places in which individuals live have been shown to influence their levels of contact with nature, and as a result, their environmental behaviour, the proximity to nature of the place of residence has clearly a strong impact on connectedness to nature. A factor that has been reported as important within the place of residence is contact with nature, closeness to nature looks to increase connectedness with nature [52] through experiences and contact [43]. This can be explained through concepts such as place attachment. Place attachment is usually defined as a positive connection or emotional bond between a person and a particular place [52, 53], definition that is closest to connectedness to nature, but addresses a connection in superior level, with the whole environment and not only the natural dimension [54]. Various studies have identified a positive correlation between place attachment and connectedness to nature, specifically the behaviour dimension, contributing on the frequency of pro-environmental actions [53–55]. Individuals can identify themselves with elements present in their places of residence, including nature, and in this way reinforce their emotional links with said contexts, manifesting behaviours oriented to the protection of said places [51, 52]. In our findings, countryside reports higher values of connectedness to nature in Spain. Rural areas have been shown to offer greater opportunities for their residents to experience contact with nature [19]. Rural residents spend more time in nature than their urban counterparts, recalling their experiences in nature as positive [56]. Hinds & Sparks [57] found that children in rural settings are more connected to nature than children in urban settings, showing more environmental attitudes and behaviour, and preference for activities on nature, this explained through the children experiences in nature. Rosa et al. [58] adds that childhood experiences and bonds with natural environments can lead to more connectedness to nature in adulthood, but there are other pathways to achieving this experiences, and not only rural settings, but also experiencing nature in urban settings.

In line with the previous, results in Ecuador shows higher values of connectedness to nature in the city. For Gifford & Nilsson [19], research from numerous countries has yielded conflicting results, specifically people in urban contexts showing more pro-environmental attitudes than people in rural settings. Arcury & Christianson [59] found that more metropolitan and urban respondents had a stronger environmental world view and were more knowledgeable about global environmental problems. Continuing with the previous, their findings don not differ from non-metropolitan and rural residents in environmental concern or environmental actions. For Kennedy et al. [60] the differences among rural and urban citizens may be diminishing since factors as migration of urban residents with pro-environmental values to rural communities and access to environmental services in rural communities can influencing the growing similarities between rural and urban populations. Restall et al. [22] finds in their research higher values of connectedness to nature in places extensively shaped by man that combine ‘artificial’ nature and anthropogenic landscape, concluding that connectedness to nature may possibly be best enhanced by providing a diversity of natural elements close to where people live and spend their day, focusing on landscapes instead of urban-rural settings.

The findings of this study have significant implications for conservation efforts and the promotion of pro-environmental behaviour in both rural and urban settings in order to avoid the potential ecological repercussions of diminished connectedness with nature. Also, for society, fostering a strong connection with nature can lead to a more environmentally conscious and proactive citizenry. A lack of engagement with nature may contribute to weakened environmental stewardship [61]. In rural contexts, where individuals often have direct and tangible interactions with nature, fostering a deeper connectedness can be leveraged to enhance local conservation initiatives. The promotion of engagement with natural environments can lead to increased pro-environmental attitudes and behaviours [62]. In urban environments, where disconnection from nature could be more prevalent, interventions should focus on creating opportunities for meaningful nature experiences. The previous underscores the importance of urban natural spaces in fostering a sense of connectedness with nature [25, 63]. Integrating natural elements into urban planning to enhance connectedness with nature has significant implications for public health and well-being. As urbanization continues to reduce opportunities of contact with nature [22], urban planners should prioritize the development and maintenance of green spaces to foster connections with nature. Green spaces can not only enhance the quality of life but also promote healthier and more environmentally conscious communities [54]. Additionally, incorporating educational programs that emphasize the importance of nature and outdoor activities can further strengthen this connection and support public health initiatives [31]. These efforts must be focused on develop the connectedness with nature on children and young adults (since the 18 to 25 years old presented lower values on our research) reinforce this connection in later stages of adulthood and reducing the gap of contact with nature opportunities between genders. Moreover, understanding the role of factors in shaping connectedness with nature is crucial. As noted by Schultz [61], tailoring conservation messages to resonate with diverse cultural backgrounds and socioeconomic contexts is essential for effective environmental advocacy. By recognizing the diverse determinants of connectedness with nature in both rural and urban contexts, conservationists, educators and policymakers can design targeted strategies to develop pro-environmental behaviours and enhance overall ecological stewardship, also, tailor environmental education programs to be more culturally relevant and effective, and promoting sustainable practices on a broader scale.

For university students, the implications are equally profound. Enhanced nature connectedness can lead to better mental health outcomes, reduced stress, and improved overall well-being, as supported by our findings. This study contributes to the growing body of evidence that highlights the importance of integrating nature into educational and urban planning strategies to achieve sustainable goals.

### Limitations and future research

The resulting data are cross-sectional and correlational, which prevents us from making conclusions regarding the meaning of relationships and causality. Although we consider our sample meets the requirements for our research, we can suggest future research a more heterogenous sample, specifically in the age and gender variable. The majority of female participants in the research can has implications for generalizability. Since our sample is composed by university students, we can justify this selection with the need for a way to measure the knowledge about environment from the career variable, that comprise various factors that contextualize connectedness with nature for the students in determined field of knowledge. Also, using data gathered from students difficult the generalizability of the results and present the possibility of bias as it is self-recorded data. However, to reproduce the present research in a larger sample, we suggest that other ways of measuring participants’ knowledge about the environment be considered.

In light of the results, our conclusions and the literature consulted, we suggest that future studies analyze the construct of connectedness with nature, focusing on the composition of the environments under study, considering the natural and built elements that compose the environments, and accessibility to these elements, addressing social norms, culture, and other normative influences that can shape pro-environmental attitudes [9]. Finally, we could suggest future lines of research in which we consider replicating the results with longitudinal studies and with a general sample and not just students. Including studies exploring interventions to enhance nature connectedness and investigating the long-term effects of nature connectedness on conservation behaviour, including methodologies that explore the underlying mechanisms through which different factors influence the connection with nature.

## Conclusions

The present research stands out due to its comparative analysis of university students’ connectedness with nature across Spain and Ecuador, offering a unique perspective on how contexts influence this relationship. Our research highlights significant differences and similarities between diverse contexts in previous research. The use of the ABC-CNS scale to analyze the multidimensional aspects of nature connectedness, provides a novel approach that enhances our understanding of the factors shaping environmental attitudes and behaviours. These findings contribute to the field of environmental education and public health, emphasizing the importance of internal and external factors in fostering a deeper connection with nature.

Following our main hypothesis, we can conclude that, indeed, the place of residence influences the levels of connectedness with nature. However, it must be considered that this influence is based on the contexts in which individuals develops, contexts that can be very particular to a country or even a region. We must understand that although contact with nature, one of the main variables to consider in connection with nature, occurs more frequently in rural than urban settings, a paradigm shift is necessary towards the analysis of the composition of environments, beyond the urban/rural classification, to understand the interaction of natural and build elements on connectedness with nature. In other words, the capacity of environments, regardless of whether they are urban or rural, to provide opportunities for contact with nature, thus raising the levels of connectedness with nature.

## Supporting information

S1 Table Mean and standard deviation of the ABC-CNS scale and each of its dimensions depending on the study variables. Detailed items are available on Cuadrado et. al [23].
(PDF)

S2 Table Mean and standard deviation of each item (in general and by country) of the ABC-CNS scale. (PDF)

S3 Table General Linear Mixed Models (GLMMs) with the affective (model 2), behavioural (model 3), and cognitive dimensions (model 4) as response variables. (PDF)

S4 Table LSD Fisher test (cognitive, affective, behaviour). Mean values with a common letter are not significantly different (p > 0.05).
(PDF)
