# Supplementary material for: Factors that determine the connectedness with nature in rural and urban contexts
Source: PLoS One. 2024 Aug 30;19(8):e0309812. doi: 10.1371/journal.pone.0309812 (PMC11364249; doi:10.1371/journal.pone.0309812)
Supplement: S1 Table — Detailed items are available on Cuadrado et. al [23]. (PDF) [file pone.0309812.s001.pdf]

S1 Table. Mean and standard deviation of the ABC-CNS scale and each of its dimensions depending on the study variables.

		Spain								Ecuador							
		ABCCNS		Cognitive		Affective		Behaviour		ABCCNS		Cognitive		Affective		Behaviour	
		Mean	Stan. Dev.	Mean	Stan. Dev.	Mean	Stan. Dev.	Mean	Stan. Dev.	Mean	Stan. Dev.	Mean	Stan. Dev.	Mean	Stan. Dev.	Mean	Stan. Dev.
All		3.94	0.63	3.90	0.78	4.09	0.73	3.84	0.68	4.15	0.81	4.05	1.03	4.29	0.93	4.12	0.80
Residence	City	3.91	0.61	3.87	0.73	4.06	0.73	3.80	0.67	4.18	0.78	4.10	0.97	4.32	0.91	4.13	0.79
	Countryside	4.28	0.37	4.17	0.67	4.49	0.41	4.17	0.52	4.16	0.81	4.04	1.04	4.29	0.93	4.15	0.77
	Town	3.98	0.68	3.93	0.86	4.11	0.75	3.91	0.70	4.06	0.90	3.92	1.15	4.20	1.01	4.08	0.83
Gender	Men	3.84	0.69	3.81	0.78	3.95	0.79	3.75	0.75	4.07	0.92	3.95	1.17	4.18	1.04	4.08	0.91
	Women	3.97	0.61	3.92	0.77	4.13	0.71	3.87	0.66	4.21	0.73	4.12	0.93	4.35	0.86	4.15	0.72
Age range	18-20	3.90	0.61	3.86	0.78	4.04	0.74	3.80	0.66	4.16	0.76	4.06	0.96	4.32	0.86	4.10	0.77
	21-25	3.94	0.64	3.88	0.77	4.11	0.69	3.84	0.70	4.12	0.89	3.99	1.13	4.22	1.02	4.14	0.85
	26-50	4.30	0.65	4.28	0.72	4.39	0.81	4.24	0.59	4.32	0.75	4.35	0.90	4.38	0.97	4.23	0.74
University	ESPAM	.	.	.	.	.	.	.	.	4.03	0.90	3.91	1.13	4.18	1.06	3.98	0.90
	ULEAM	.	.	.	.	.	.	.	.	4.07	0.83	3.96	1.07	4.18	0.99	4.06	0.83
	UNESUM	.	.	.	.	.	.	.	.	4.33	0.88	4.19	1.15	4.47	0.98	4.32	0.82
	UTM	.	.	.	.	.	.	.	.	4.25	0.70	4.17	0.87	4.39	0.78	4.19	0.69
	UCO	3.96	0.63	3.92	0.76	4.09	0.73	3.85	0.68	.	.	.	.	.	.	.	.
	USAL	3.69	0.66	3.36	0.91	4.04	0.70	3.66	0.63	.	.	.	.	.	.	.	.
Career	Engineering. industry and administration	.	.	.	.	.	.	.	.	4.09	0.85	3.98	1.08	4.22	1.00	4.05	0.85
	Environment And biology	4.05	0.58	4.04	0.72	4.18	0.69	3.94	0.62	4.21	0.84	4.10	1.08	4.35	0.94	4.19	0.84
	Psychology. education and social sciences	3.91	0.64	3.86	0.79	4.06	0.74	3.82	0.69	4.17	0.74	4.09	0.91	4.29	0.85	4.13	0.69
