# Supplementary material for: Factors that determine the connectedness with nature in rural and urban contexts
Source: PLoS One. 2024 Aug 30;19(8):e0309812. doi: 10.1371/journal.pone.0309812 (PMC11364249; doi:10.1371/journal.pone.0309812)
Supplement: S2 Table — (PDF) [file pone.0309812.s002.pdf]

S2 Table. Mean and standard deviation of each item (in general and by country) of the ABC-CNS scale. Detailed items are available on Cuadrado et. al [23].

	General		Spain		Ecuador	
	Mean	Stan. dev.	Mean	Stan. dev.	Mean	Stan. dev.
**Cognitive.**						
CNS1.	4.19	1.03	4.30	0.85	4.12	1.12
CNS2.	4.00	1.04	3.83	0.92	4.10	1.09
CNS3.	3.91	1.04	3.78	0.91	3.99	1.10
CNS4.	3.81	1.10	3.48	1.01	4.00	1.10
CNS5.	4.07	1.05	4.09	0.96	4.06	1.10
**Affective**						
CNS6.	4.34	0.98	4.37	0.84	4.32	1.06
CNS7.	4.15	0.98	3.97	0.89	4.25	1.01
CNS8.	4.04	1.01	3.73	0.98	4.22	0.98
CNS9.	4.16	0.97	3.95	0.91	4.28	0.99
CNS10.	4.38	0.92	4.41	0.78	4.37	0.99
**Behavioral**						
CNS11.	3.92	0.99	3.73	1.00	4.03	0.96
CNS12.	3.81	1.00	3.45	0.98	4.02	0.94
CNS13.	4.03	0.91	3.91	0.90	4.09	0.91
CNS14.	4.08	0.90	3.90	0.95	4.18	0.86
CNS15.	4.27	0.92	4.23	0.98	4.29	0.89
