# Supplementary material for: Factors that determine the connectedness with nature in rural and urban contexts
Source: PLoS One. 2024 Aug 30;19(8):e0309812. doi: 10.1371/journal.pone.0309812 (PMC11364249; doi:10.1371/journal.pone.0309812)
Supplement: S3 Table — (PDF) [file pone.0309812.s003.pdf]

S3 Table. General Linear Mixed Models (GLMMs) with the affective (model 2), behavioural (model 3), and cognitive dimensions (model 4) as response variables.

		Marginal			
		F-value	p-value		Value ± S.E.
**MODEL II**	Intercept	937.00	<0.0001	Intercept	3.85 ± 0.15
**COGNITIVE**	Gender	11.04	0.0009	Women	0.10 ± 0.07
	Age range	7.95	0.0004	Age range 21-25<br>Age range 26-50	-4.9E-03 ± 0.06<br>0.38 ± 0.10
	Career	2.67	0.0697	Environment and biology<br>Psychology. education and social sciences	0.11 ± 0.09<br>-0.06 ± 0.09
	Place of residence	3.53	0.0295	Countryside<br>Town	0.02 ± 0.14<br>-0.36 ± 0.12
	Gender:Place of residence	3.75	0.0238	Women:Countryside<br>Women:Town	-2.2E-04 ± 0.16<br>0.36 ± 0.14
**MODEL III**	Intercept	2150.66	<0.0001	Intercept	4.09 ± 0.12
**AFFECTIVE**	Gender	11.80	0.0006	Women	0.15 ± 0.06
	Age range	2.91	0.0551	Age range 21-25<br>Age range 26-50	-0.01 ± 0.05<br>0.20 ± 0.09
	Career	3.97	0.0190	Environment and biology<br>Psychology. education and social sciences	0.14 ± 0.08<br>-0.04 ± 0.08
	Place of residence	2.90	0.0554	Countryside<br>Town	0.12 ± 0.13<br>-0.27 ± 0.11
	Gender:Place of residence	3.59	0.0278	Women:Countryside<br>Women:Town	-0.11 ± 0.15<br>0.30 ± 0.13
**MODEL IV**	Intercept	805.59	<0.0001	Intercept	3.85 ± 0.16
**BEHAVIOUR**	Gender	3.70	0.0547	Women	0.09 ± 0.06
	Age range	5.79	0.0031	Age range 21-25<br>Age range 26-50	0.06 ± 0.05<br>0.27 ± 0.08

Career	3.03	0.0487	Environment and biology<br>Psychology. education and social sciences	0.10 ± 0.07<br>-0.03 ± 0.07
Place of residence	2.35	0.0957	Countryside<br>Town	0.20 ± 0.11<br>-0.10 ± 0.09
Gender:Place of residence	2.16	0.1152	Women:Countryside<br>Women:Town	-0.13 ± 0.13<br>0.17 ± 0.11

Reference levels:
Gender: Men
Age range: 18-20
Career: Engineering. industry, and administration
Place of residence: City
