# Supplementary material for: Factors that determine the connectedness with nature in rural and urban contexts
Source: PLoS One. 2024 Aug 30;19(8):e0309812. doi: 10.1371/journal.pone.0309812 (PMC11364249; doi:10.1371/journal.pone.0309812)
Supplement: S4 Table — Mean values with a common letter are not significantly different (p > 0.05). (PDF) [file pone.0309812.s004.pdf]

S4 Table. LSD Fisher test (Cognitive, Affective, Behaviour). Mean values with a common letter are not significantly different (p > 0.05).

		Cognitive				Affective					Behaviour			
		Mean	S.E.			Mean	S.E.				Mean	S.E.		
Gender	Women	4.13	0.09	A		4.37	0.07	A			4.16	0.08	A	
	Men	3.91	0.10		B	4.15	0.08		B		4.06	0.09	A	
Age range	26-50	4.27	0.13	A		4.40	0.11	A			4.27	0.10	A	
	21-25	3.89	0.09		B	4.18	0.07		B		4.06	0.08		B
	18-20	3.89	0.09		B	4.19	0.07		B		4.00	0.08		B
Career	Environment and biology	4.11	0.10	A		4.36	0.08	A			4.19	0.09	A	
	Engineering, industry and ..	4.01	0.11	A	B	4.23	0.09	A	B		4.11	0.09	A	B
	Psychology, education and ..	3.94	0.10		B	4.18	0.08		B		4.04	0.08		B
Residence	Countryside	4.09	0.11	A		4.35	0.09	A			4.21	0.10	A	
	City	4.07	0.09	A		4.28	0.07	A			4.07	0.08		B
	Town	3.89	0.10		B	4.15	0.08		B		4.05	0.09		B
Gender:Residence	Women:Countryside	4.14	0.12	A		4.37	0.10	A	B		4.19	0.10	A	
	Women:Town	4.13	0.10	A		4.37	0.08	A			4.18	0.09	A	
	Men:Countryside	4.04	0.15	A		4.33	0.13	A	B		4.23	0.12	A	
	Women:City	4.12	0.09	A		4.35	0.07	A	B		4.11	0.08	A	
	Men:City	4.02	0.10	A		4.20	0.08		B		4.03	0.08	A	B
	Men:Town	3.66	0.13		B	3.93	0.11			C	3.92	0.11		B
